# Interactive effects of herbivory and substrate orientation on algal community dynamics on a coral reef

**DOI:** 10.1007/s00227-018-3411-2

**Published:** 2018-09-14

**Authors:** Alain Duran, L. Collado-Vides, L. Palma, D. E. Burkepile

**Affiliations:** 1 Department of Biological Sciences, Florida International University, 11200 S.W. 8th St., Miami, FL 33199 USA; 2 Center for Coastal Oceans Research in the Institute for Water and Environment, 11200 S. W. 8th St., Miami, FL 33199 USA; 3 Department of Ecology, Evolution and Marine Biology, University of California, Santa Barbara, Santa Barbara, CA 93106 USA; 4 Marine Science Institute, University of California, Santa Barbara, Santa Barbara, CA 93106 USA

## Abstract

Herbivory is a significant driver of algal community dynamics on coral reefs. However, abiotic factors such as the complexity and orientation of the benthos often mediate the impact of herbivores on benthic communities. We experimentally evaluated the independent and interactive effects of substrate orientation and herbivorous fishes on algal community dynamics on a coral reef in the Florida Keys, USA. We created horizontal and vertical substrates, mimicking the trend in the reduction of vertical surfaces of coral reefs, to assess how algal communities developed either with herbivory (open areas) or without herbivory (herbivore exclosures). We found that substrate orientation was the dominant influence on macroalgal community composition. Herbivores had little impact on community development of vertical substrates as crustose algae dominated these substrates regardless of being in exclosures or open areas. In contrast, herbivores strongly impacted communities on horizontal substrates, with upright macroalgae (e.g., *Dictyota* spp., articulated coralline algae) dominating herbivore exclosures, while filamentous turf algae and sediment dominated open areas. Outside of exclosures, differences between vertical and horizontal substrates exposed to herbivores persisted despite similar intensity of herbivory. Our results suggest that the orientation of the reef benthos has an important impact on benthic communities. On vertical surfaces, abiotic factors may be more important for structuring algal communities while herbivory may be more important for controlling algal dynamics in flatter areas. Thus, the decline in structural complexity of Caribbean coral reefs and the flattening of reef substrates may fundamentally alter the impact that herbivores have on benthic community dynamics.

**Electronic supplementary material:**

The online version of this article (10.1007/s00227-018-3411-2) contains supplementary material, which is available to authorized users.

## Introduction

Herbivory is a fundamental process on coral reefs that regulates algal species composition, algal abundance (Ogden and Lobel 1978; Lewis and Wainwright 1985; Carpenter 1986) and the interactions between corals and algae (Mapstone et al. 2007; Trapon et al. 2013a, b; Zaneveld et al. 2016). On Caribbean coral reefs, sea urchins like the long-spined sea urchin (*Diadema antillarum*) and herbivorous fishes [Family Acanthuridae (surgeonfishes) and Family Labridae, tribe Scarini (parrotfishes)] are often the most abundant herbivores (Steneck 1983; Adam et al. 2015a). A die-off of sea urchins in the 1980s left fishes as the main herbivores on Caribbean reefs (Lessios 1988), although they are currently overfished in many areas (Jackson et al. 2014). Reduced herbivory and concurrent declines in coral cover have facilitated increases in macroalgal cover, which has doubled Caribbean-wide since the 1970s (Jackson et al. 2014). Declines in coral abundance coupled with increased bioerosion rates have resulted in an overall negative carbon budget on many reefs, driving reductions of structural complexity on many reefs throughout the region (Alvares-Filip et al. 2011; Perry et al. 2014).

The structural complexity of coral reefs largely comprised the three-dimensional physical structure built by scleractinian corals and other calcifying organisms that provide shelter, settlement opportunities, and foraging habitat to reef-dwelling organisms (Wilson et al. 2007; Graham and Nash 2013). Areas with higher structural complexity often have more abundant sea urchins (Fabricius et al. 2014) and herbivorous fishes (Luckhurst and Luckhurst 1978; Graham 2014; Rogers et al. 2014), which may increase top-down control on algal communities (Verges et al. 2011). For instance, crustose coralline algae (CCA), which commonly dominate areas with high herbivory (Steneck 1997), are often more abundant on reefs with higher complexity and more steeply sloped substrates (Fabricius and De’ath 2001). However, in the Caribbean, the structural complexity of coral reefs has declined by more than 50% since the 1960s, creating flatter, more horizontal reef surfaces (Alvares-Filip et al. 2009, 2011). Thus, there is a critical need to understand how the loss of structural complexity and the flattening of coral reefs influence herbivory and algal community dynamics.

Reduced structural complexity on reefs could alter herbivory, and consequently algal dynamics, through several mechanisms (Bozec et al. 2013, 2015). For instance, lower complexity reefs provide less shelter for herbivorous fishes, which may reduce herbivory and result in more macroalgae (Verges et al. 2011). Additionally, more complex reefs may require more grazing pressure as a consequence of having more area that needs to be grazed by herbivores (Bozec et al. 2013). At a smaller scale, microtopographic complexity often influences grazing dynamics by giving differential access to different species of herbivorous fishes, which alters the diversity of algae and abundance of juvenile corals on the scale of centimeters (Brock 1979; Brandl and Bellwood 2016). Furthermore, the flattening of reef substrates could increase sediment accumulation, which, in turn, can reduce grazing activity and promote the growth of filamentous algae (Goatley and Bellwood 2013; Clausing et al. 2014). Conversely, benthic areas with steeper slopes tend to have less sediment, which may facilitate herbivory and the abundance of CCA, including species [e.g., *Titanoderma prototypum* (Foslie) Woelkerling, Y.M. Chamberlain and P.C. Silva] that can facilitate coral recruitment (Arnold and Steneck 2010; Ritson-Williams et al. 2016). Therefore, the ongoing flattening of Caribbean coral reefs may have a strong impact on herbivores and their role as drivers of algal dynamics.

Here, we investigated how structural complexity can mediate the influence of herbivory on algal community dynamics on a reef in the Florida Keys, USA. We manipulated the orientation (horizontal vs. vertical) of experimental substrates using quarried coral limestone tiles to simulate bare substrate created after a disturbance. To examine the interaction between substrate orientation and herbivory, we established these substrates in areas with low (herbivore exclosures) or high (open areas) herbivory. We expected that substrate orientation would determine whether herbivores strongly impact the dynamics of benthic macroalgae. We predicted that herbivory would strongly impact algal communities on horizontal substrates with filamentous turf algae dominating in open areas and upright macroalgae dominating in exclosures as herbivores tend to retard algal succession (Diaz-Pulido and McCook 2002; Duran et al. 2016). On vertical substrates, we expected that herbivory would be less important, possibly as a consequence of reduced light (Roff et al. 2015), such that crustose algae would dominate vertical surfaces in open areas and in herbivore exclosures.

## Materials and methods

### Study site

We conducted our experiment from August 2013 to August 2014 on a low relief spur and groove reef near Conch Reef (24°57.695′W, 80°27.230′N) in ~ 7 m of water located in the upper Florida Keys, USA. These reefs are regularly dominated by turf algae with seasonal peaks of *Stypopodium zonale* in the spring months and *Dictyota* spp. in the summer months (Zaneveld et al. 2016). Reefs in the Florida Keys have very low sea urchin density (< 0.1 Ind. m^−2^; Chiappone et al. 2008) and high abundance of herbivorous fishes (Burkepile et al. 2013) including large parrotfishes currently considered rare Caribbean-wide (Adam et al. 2015b).

### Experimental manipulation

**Fig. 1:**
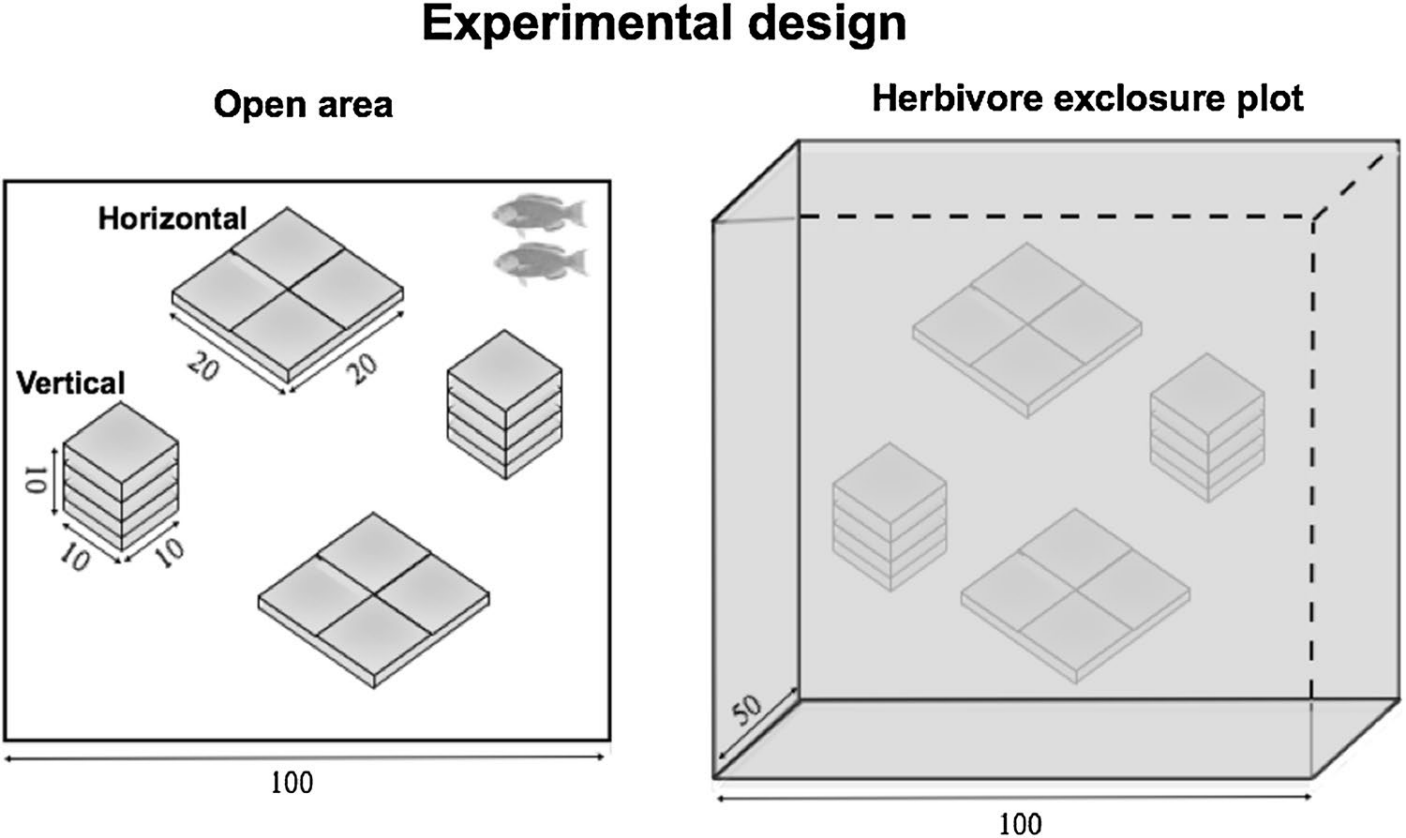
Experimental design showing the dimensions of substrates in open areas (left) and herbivore exclosures (right). Notice that each vertical substrate encompasses four (10 × 10 cm) vertical walls that are the same area as horizontal substrates (20 × 20 cm = 400 cm^2^). Numbers indicate the dimensions in cm. *n *= 3 for each open area and herbivore exclosure

We used quarried coral limestone tiles (2.5 × 10 × 10 cm) to create bare vertical and horizontal substrates that mimic areas of reef with distinct substrate orientation (Fig. 1). We assembled four tiles next to each other to construct flat squares (20 × 20 cm; 400 cm^2^ total area) of horizontal substrate and four tiles stacked to create vertical substrates (10 × 10 cm per side for 400 cm^2^ total area by adding all four sides; Fig. 1b). We did not include the horizontal surface on the top of the vertical tiles in any of the data collection to keep the area of the treatments the same. Because of the nature of this setup, the horizontal substrates had 40 cm of cracks, spaces where tiles met each other, while the vertical tiles had 120 cm of cracks. Thus, we only collected data on the exposed tile surfaces, not on the organisms growing within the cracks, to prevent this difference from confounding our quantification of the benthic communities.

In order to test the impact of herbivory on algal communities, we placed two sets of each substrate type inside an exclosure plot (1 × 1 × 0.5 m). Exclosures were framed with stainless steel round bar and covered with PVC-coated wire mesh with 2.5 cm diameter holes to exclude herbivorous fishes (2 horizontal substrates and 2 vertical substrates within one exclosure). Another two sets of each substrate orientation were placed in each open area with open access to herbivorous fishes. Thus, the substrate orientation treatment was nested within either exclosures or open areas, creating four treatments: (1) horizontal substrates in open areas, (2) vertical substrates in open areas, (3) horizontal substrates in exclosures, and (4) vertical substrates in exclosures. Each of these treatments was replicated three times (*n *= 3 exclosures and *n *= 3 open areas). All substrates were deployed in August 2013 and data collection began in September 2013 and continued until August 2014.

We did not include exclosure controls in our experiment as previous research suggested minimal effects of exclosure artifacts on algal communities in these shallow reef systems (Miller et al. 1999; Smith et al. 2001; Burkepile and Hay 2007). In fact, our recent study on a nearby reef showed no effects of exclosures on water flow, sedimentation, or algal communities using similarly designed exclosures (Zaneveld et al. 2016; see supplementary material therein for a discussion of potential exclosure artifacts). However, these exclosures do decrease light availability to the benthos by ~ 15% (Ferrari et al. 2012). Given that the light availability common at these shallow depths saturates the photosystems of most primary producers (Carpenter 1985), the slight decrease in light availability likely had minimal impact on primary production or interactions among benthic organisms.

### Herbivorous fish feeding

We recorded the grazing activity of parrotfishes and surgeonfishes on vertical and horizontal substrates in open areas using GoPro video cameras. Grazing activity was evaluated six times during the experiment: in September 2013, October 2013, December 2013, February 2014, April 2014, and May 2014. Cameras were placed 50 cm away from each plot between 1000 and 1400 hours to film grazing activity on both horizontal and vertical substrates simultaneously. To quantify grazing intensity on each substrate we selected 20 random 5-min periods from the 3 to 4 h of video during each deployment. We identified every fish that fed on the substrates to species and recorded life history stage (juvenile, intermediate, adult), as well as the type of substrate bitten (horizontal vs. vertical), and the number of bites during each feeding event. We did not include bites on the top of the vertical substrates (the flat horizontal portion on the top of the stack of tiles) to ensure we quantified bites in the same area on both vertical and horizontal substrates.

### Algal community dynamics

Every 30–45 days between August 2013 and August 2014 (*n *= 8 sampling periods) we visually surveyed the benthic community on the vertical and horizontal substrates. To do so, we placed a 10 × 10 cm grid divided into four quadrants over the substrate and visually estimated the percent of the substrate covered by different algal taxa to the nearest 5%. We identified algae to the lowest taxonomic level possible and also grouped them into form–functional groups (FFG) following a modification of Steneck and Dethier (1994). We considered turf algae (hereafter “turf”) as all short (< 1 cm) filamentous algal species with little to no sediment trapped in the filaments (Connell et al. 2014). When these filamentous algal communities became longer (> 2 cm height), they often trapped sediment within the filaments. Therefore, we classified this matrix as turf algae, following Connell et al. (2014) for the definition of turf, associated with sediment (henceforth “TAS” or ‘turf and associated sediment’). When sediment was on the substrate but not associated with turf algae, it was classified as sediment.

### Statistical analysis

We evaluated the effect of substrate orientation and month on herbivore grazing rates via Friedman tests. Unfortunately, no transformations were able to make the grazing rate data normal, and we had to resort to nonparametric statistics. We transformed the benthic percent cover data via Box–Cox transformations to meet assumptions of homoscedasticity and normality (as checked for using the Levene’s test). After transformation, we used a linear mixed model (LMM) to test the effects of herbivory and substrate orientation over time (month) for each benthic group with plot as a random factor. We assessed changes in community composition through time for each treatment using non-metric multidimensional scaling (NMDS) analyses and permutational multivariate analysis of variance (PERMANOVA) with the distance matrix calculated using Bray–Curtis dissimilarity. We performed descriptive and inferential analyses using packages vegan (Oksanen et al. 2017), doBy (Soren 2016), MASS (Venables and Ripley 2002), ggplot2 (Wickham 2009) in the R program created by R Development Core Team (2016), version, 3.2.2.

## Results

### Herbivorous fish feeding

**Fig. 2:**
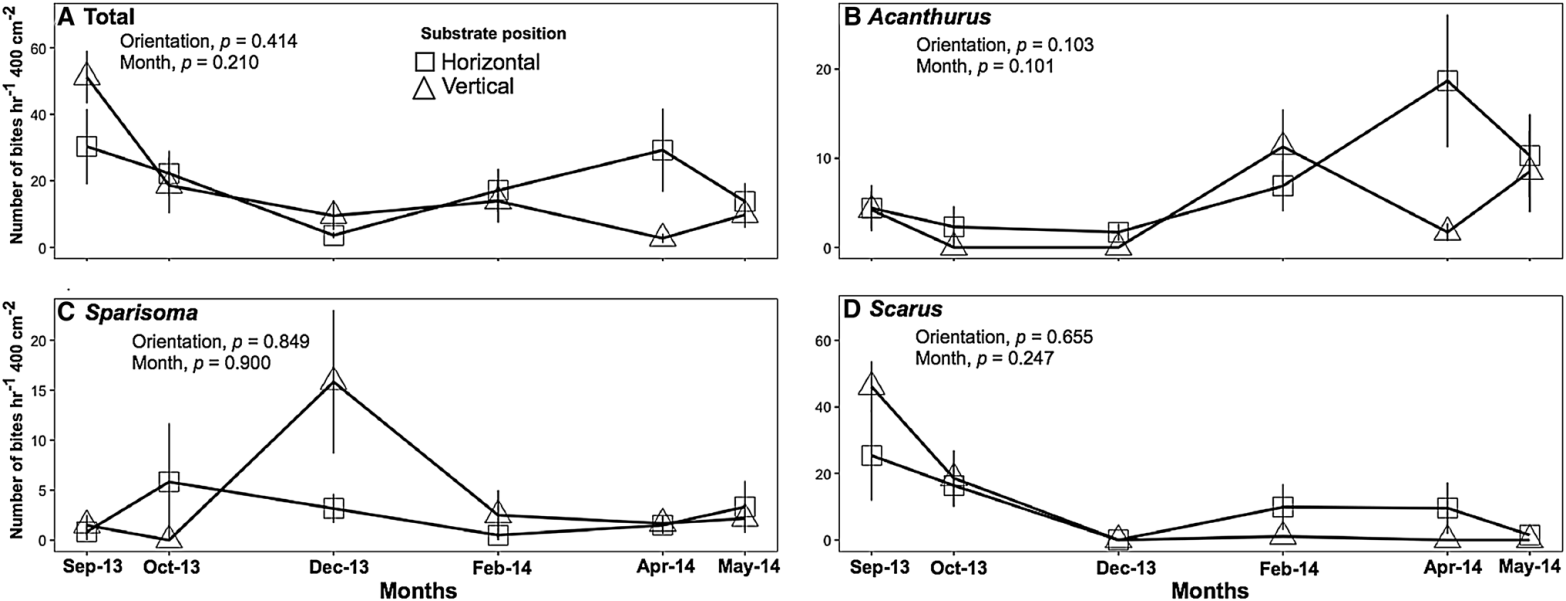
Grazing rates by herbivorous fishes obtained from videos recorded in open areas. Points represent the number of bites taken by **a** all species of herbivorous fishes, **b** *Acanthurus* spp., **c** *Sparisoma* spp., or **d** *Scarus* spp. Data are mean ± SE. Note that *Y* axes vary in scale. Statistics are from Friedman tests

Overall grazing rates of herbivores did not differ across time or between substrate orientation (horizontal or vertical) (Fig. 2a). Grazing rates by surgeonfishes were similar on both substrate orientations with an average of 7.4 ± 2.0 bites h^−1^ 400 cm^−2^ on horizontal substrates and 4.5 ± 1.5 bites h^−1^ 400 cm^−2^ on vertical substrates (Fig. 2b). While *Sparisoma* spp. and *Scarus* spp. parrotfishes took approximately 2 and 10 bites h^−1^ 400 cm^−2^, respectively, neither genus exhibited preferences for a specific substrate orientation (Fig. 2c, d).

### Algal community dynamics

**Fig. 3:**
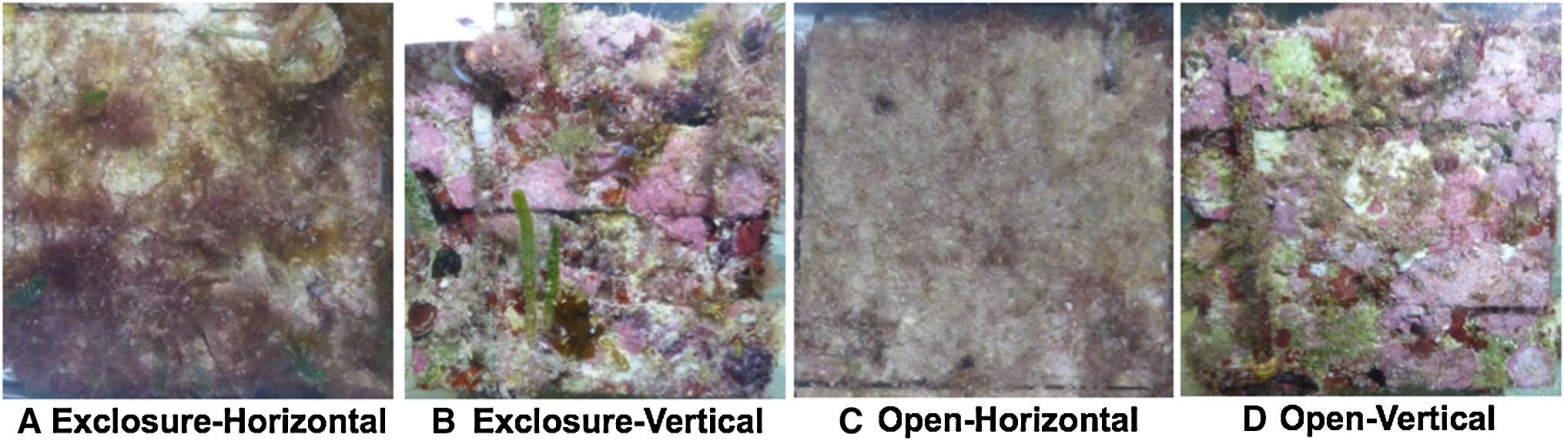
Photographs of community composition on experimental substrates at the end of the year-long experiment: **a** exclosure-horizontal, **b** exclosure-vertical, **c** open-horizontal, and **d** open-vertical substrates. Photos were taken on 10 × 10 cm section of the substrates

**Fig. 4:**
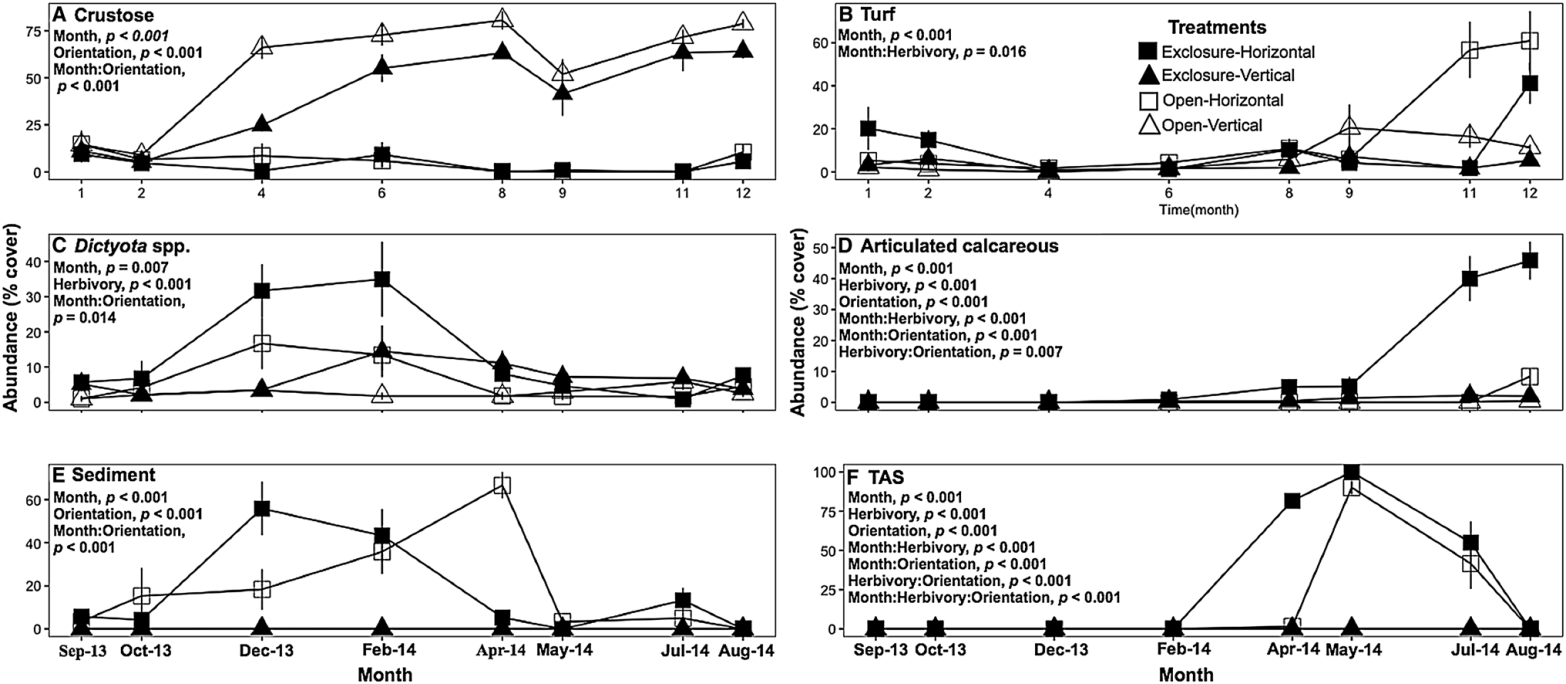
Abundance of different benthic groups through time in different treatments: horizontal substrates in herbivore exclosures (exclosure-horizontal), vertical substrates in herbivore exclosures (exclosure-vertical), horizontal substrates in open areas (open-horizontal) and vertical substrates in open areas (open-vertical). **a** Crustose algae, **b** turf algae, **c** *Dictyota* spp., **d** articulated calcareous algae, **e** sediment and **f** turf associated with sediment (TAS). Data are mean ± SE. Note that *Y* axes vary in scale. Statistics show significant effects from linear mixed models. See supplementary material for full model outputs

Substrate orientation was a strong determinant of algal community composition with horizontal and vertical substrates often differing regardless of herbivory (Figs. 3, 4). On vertical substrates, crustose algae covered more than 50% of the substrate after 6 months and remained the dominant benthic group regardless of herbivory treatment (Fig. 4a, see Appendix I in ESM for complete model results). Turf algae were often the second most abundant algal group on vertical substrates ranging from 3.4 ± 0.6 to 7.3 ± 1.8% in exclosures and open treatments, respectively, with marginal differences between orientations (Fig. 4b, LMM, Orientation, *F*_1,62_ = 3.7, *p* = 0.059). Upright macroalgae (*Dictyota* spp. and articulated calcareous algae), sediment, and TAS were rarely present on vertical substrates throughout the entire study regardless of herbivory (Fig. 4c–f).

Herbivory more strongly impacted algal communities on the horizontal substrates. On horizontal substrates in open areas, turf abundance remained below 25% for the first 9 months after which turf cover sharply increased to more than 50% (Fig. 4b, LMM, Month:Herbivory, *F*_7,62_ = 2.7, *p* = 0.016, see Appendix I in ESM for complete model results). Macroalgal abundance on horizontal substrates varied through time depending on the presence of herbivory. *Dictyota* dominated horizontal substrates in exclosures during early succession with a peak of ~ 35% in February 2014 followed by a drop of abundance to less than 10% (Fig. 4c, LMM, month:orientation, *F*_7,62_ = 4.8, *p* < 0.001). Turf associated with sediment (TAS) developed on horizontal substrates after a previous accumulation of sediment on the substrates (Fig. 4e, f). By May 2014, TAS was the most abundant group on horizontal substrates covering close to 100% of the substrate (Fig. 4f, LMM, orientation, *F*_1,62_ = 414.1, *p* < 0.001; month, *F*_7,62_ = 106.8, *p* < 0.001). However, after the peak in TAS on horizontal substrates, articulated calcareous algae (e.g., *Amphiroa* spp. and *Jania* spp.) became the dominant macroalgal group on horizontal substrates in herbivore exclosures, covering over 45% of the substrate by the end of the experiment (Fig. 4d, LMM, month, herbivory:orientation, *F*_1,62_ = 7.9, *p* = 0.007). Articulated calcareous algae were rare on horizontal tiles exposed to herbivores.

**Fig. 5:**
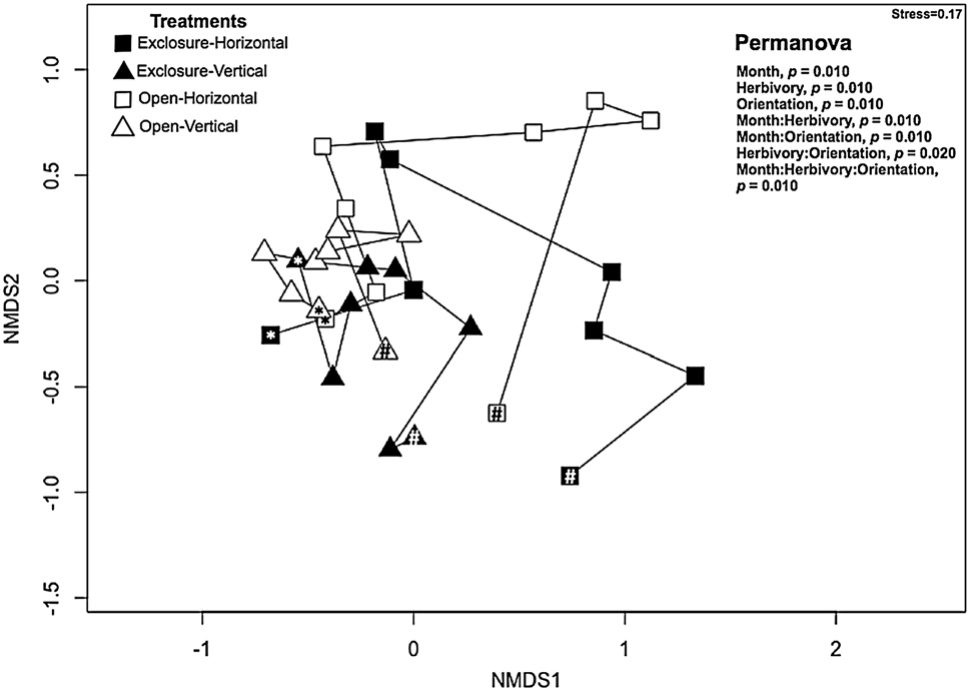
Trajectory of macroalgal community composition using non-metric multidimensional scaling (NMDS). The treatments represent: horizontal substrates in herbivore exclosures (exclosure-horizontal), vertical substrates in herbivore exclosures (exclosure-vertical), horizontal substrates in open areas (open-horizontal) and vertical substrates in open areas (open-vertical). Asterisk (*) represents the first time point after the benthic community developed (month 1) and pound symbol (#) indicates the final time point. Statistics show significant effects from PERMANOVA. See supplementary material for full model outputs

When we assessed the overall composition of macroalgal communities, both substrate orientation and herbivory led to differences in community composition over time (Fig. 5, see Appendix I in ESM for complete model results). However, substrate orientation explained the highest proportion of change in algal community (PERMANOVA, *R*^2^ = 0.19) compared to herbivory (PERMANOVA, *R*^2^ = 0.02) and showed a significant interaction with time (PERMANOVA, month:orientation, *F*_1,191_ = 12.31, *p* = 0.010). The NMDS suggested that the algal communities on vertical substrates followed similar temporal patterns regardless of herbivory. However, on horizontal substrates, herbivory appeared to drive a divergence of algal communities over time.

## Discussion

The extent to which the loss of structural complexity on coral reefs mediates the influence of herbivory on benthic communities is an important, yet minimally addressed topic. Here, we show that substrate orientation is a key driver of algal community dynamics. We found that vertical substrates were primarily dominated by crustose algae with little upright macroalgae, regardless of the presence of herbivorous fishes. In contrast, upright macroalgae such as *Dictyota* spp. and articulated calcareous algae dominated horizontal substrates when herbivorous fishes were absent. However, even in the presence of herbivores, filamentous turf algae and sediments dominated horizontal substrates in contrast to the crustose algae that dominated vertical substrates. These distinct differences in algal communities on vertical and horizontal substrates persisted despite similar intensity of herbivory on both orientations.

Several different abiotic and biotic factors may influence the impact of substrate orientation on macroalgal community composition. Vertical substrates are less likely to accumulate sediment, which can slow growth rates of CCA and reduce their abundance (Steneck 1997; Fabricius and De’ath 2001). Therefore, the lack of sediment on vertical tiles likely facilitated crustose algae, which was in stark contrast to high sediment loads (up to 60% cover during some periods) and low CCA cover on horizontal substrates. High sediment cover on horizontal substrates likely enhanced the retention and growth of new algal propagules (Steneck 1997) and facilitated the formation of a matrix of turf and associated sediment (TAS). Indeed, we observed that after 8 months of sediment accumulation on horizontal substrates, there was an increase of TAS to more than 75% cover, followed by an increased abundance of turf in open areas. The dominance of turf algae on horizontal substrates in open areas may be a function of accumulated sediment protecting turf forming algae from consumption by herbivorous fishes (e.g., Goatley and Bellwood 2013; Clausing et al. 2014; Gordon et al. 2016; Tebbett et al. 2017), although we did not see any obvious suppression of herbivory as sediment accumulated.

In contrast, reductions in herbivory strongly impacted the dynamics of algae on horizontal substrates within exclosure treatments. Herbivory often controls algal succession, with macroalgae increasing rapidly when herbivores are absent (Smith et al. 2010; Duran et al. 2016; Zaneveld et al. 2016). Our results corroborate previous findings, as horizontal substrates exposed to herbivores consistently had low cover of macroalgae and high cover of filamentous turf algae that are adapted to environments with intense grazing from herbivores (Carpenter 1986). Yet, in herbivore exclosures, macroalgae, particularly articulated calcareous algae that are typically rare where herbivory is high (e.g., Zaneveld et al. 2016), replaced turf algae over time. In contrast, herbivory had no effect on algal communities growing on vertical substrates as crustose algae dominated these substrates regardless of exclosure status. Crustose algae, potentially facilitated by their ability to proliferate under lower light conditions on the vertical substrates, are often well defended against herbivores by their crustose thallus (Steneck and Dethier 1994). In fact, herbivores often facilitate crustose algae by removing upright algae that would otherwise outcompete crustose taxa (Smith et al. 2010). Combined, our results suggest that the slope of reef habitats can strongly influence benthic community composition and regulate the importance of herbivory for structuring algal communities, at least at small spatial scales.

Although we did not measure light levels in our experiment, light exposure on vertical tiles was likely significantly lower compared to horizontal substrates as similar experiments have shown (Strader et al. 2015). These differences in light intensity could have influenced the differences in algal composition found between the two substrates. Lower light levels can facilitate certain CCA species (e.g., *Titanoderma* sp.) in crevices on shallow reefs or exposed areas on deeper reefs (Steneck and Dethier 1994). Although some CCA species (e.g., *Porolithon* sp. and *Lithophyllum* sp.) can dominate shallow areas with high light exposure (Steneck 1986; Littler and Littler 2013; Dean et al. 2015), higher light intensity can also reduce the growth of some CCA via photoinhibition (Burdett et al. 2014). The higher light intensity on the horizontal substrates combined with the inhibitory effects of increased sedimentation may have made horizontal substrates more conducive for the growth of non-crustose algae such as filamentous algae and contributed to the differences in algal communities we observed (Cheroske et al. 2000; Trapon et al. 2013a, b).

The structural complexity of reefs is often positively related to coral cover (Alvares-Filip et al. 2009; Graham and Nash 2013), but fewer studies have looked at how small-scale (< 500 cm^2^) habitat characteristics (reef complexity or substrate orientation) could influence important reef processes such as coral recruitment and species competition (Brock 1979; Vermeij 2006; Doropoulos et al. 2016; Mallela 2018). For instance, complexity of microtopographic refuges can promote coral recruitment by increasing turbulent flow and facilitating the arrival of coral larvae to the substrate (Hata et al. 2017) and by providing microrefuges (< 1000 μm) where coral larvae prefer to settle (Whalan et al. 2015). Microtopography could also mediate the grazing pressure of herbivores, especially in upward-facing surfaces, and thus their impact over benthic communities promoting local benthic diversity and increasing coral recruitment (Edmunds et al. 2014; Brandl and Bellwood. 2016). Davies et al. (2013) observed that despite high species-specific variation, corals recruited twice as much on vertical substrates compared to horizontal substrates and that corals also displayed lower mortality on vertical substrates.

Thus, the differences in algal communities between horizontal and vertical substrates that we saw could have a significant impact on the recruitment of corals (Trapon et al. 2013a, b). The abundant sediment and macroalgae on horizontal substrates would likely represent poor habitats for coral recruitment (Birrell et al. 2005). In contrast, some species of crustose coralline algae, which were abundant on vertical substrates regardless of herbivory, are strong facilitators of coral recruitment (Littler and Littler 2013; Ritson-Williams et al. 2016). In fact, the few coral recruits that we found during our experiment were only on vertical substrates with abundant crustose algae (photos in Supplementary section II). Our observations suggest that the ongoing reduction of structural complexity in the Caribbean might negatively impact coral recruitment via alterations to the benthic communities and the decline in important microhabitats that facilitate recruitment.

Most of the work investigating the impact of structural complexity on coral reef dynamics has focused on its influence on fish community composition, and the behavior and recruitment of mobile species, particularly fishes (Holbrook et al. 2000; Verges et al. 2011; Catano et al. 2015, 2016). Our data demonstrate that the flattening of reefs at a small scale can significantly influence how herbivores control macroalgal communities. Therefore, the gradual decline in structural complexity on coral reefs (Alvares-Filip et al. 2011) may significantly impact how herbivory impacts benthic community dynamics, which could contribute to important negative feedbacks detracting from the resilience of coral reefs. These differences in how herbivory impacts reef areas of different substrate orientations will be important to incorporate into future modeling efforts aimed at understanding how the loss of complexity impacts reef resilience (e.g., Blackwood et al. 2012; Bozec et al. 2015).

## Electronic supplementary material

Below is the link to the electronic supplementary material.

Supplementary material 1 (PDF 96 kb)

Supplementary material 2 (PDF 672 kb)
