# Supplementary material for: Interactive effects of herbivory and substrate orientation on algal community dynamics on a coral reef
Source: Mar Biol. 2018 Sep 14;165(10):156. doi: 10.1007/s00227-018-3411-2 (PMC6153878; doi:10.1007/s00227-018-3411-2)
Supplement: Supplementary file 1 — Supplementary material 1 (PDF 96 kb) [file 227_2018_3411_MOESM1_ESM.pdf]

Title: Interactive effects of herbivory and substrate orientation on algal community dynamics on a coral reef

Authors: A. Duran, L. Collado-Vides, L. Palma, and D. E. Burkepile

Appendix I. Statistical analyses

Analysis of herbivory rates on experimental substrates - Friedman rank sum tests

Total number of bites

	Df	chi-squared	*p*
**Orientation**	1	0.67	0.414
**Month**	5	7.14	0.210

Number of bites by *Acanthurus* spp.

	Df	chi-squared	*p*
**Orientation**	1	2.67	0.103
**Month**	5	9.20	0.101

Number of bites by *Sparisoma* spp.

	Df	chi-squared	*p*
**Orientation**	1	2.00	0.849
**Month**	5	0.10	0.900

Number of bites by *Scarus* spp.

	Df	chi-squared	*p*
**Orientation**	1	0.20	0.655
**Month**	5	6.67	0.247

Abundance of different algal groups on experimental substrates- Linear mixed models

Crustose algae

	num DF	den DF	F	*p*	
(Intercept)	1	62	102.39	<0.001	
Month	7	62	5.07	<0.001	***
Herbivory	1	62	0.86	0.358	
Orientation	1	62	260.98	<0.001	***
Month:Herbivory	7	62	0.64	0.724	
Month:Orientation	7	62	11.99	<0.001	***
Herbivory:Orientation	1	62	0.04	0.851	
Month:Herbivory:Orientation	7	62	0.48	0.844	

Turf

	num DF	den DF	F	*p*	
(Intercept)	1	62	214.18	<0.001	***
Month	7	62	6.97	<0.001	***
Herbivory	1	62	0.47	0.496	
Orientation	1	62	3.71	0.059	
Month:Herbivory	7	62	2.71	0.016	**
Month:Orientation	7	62	1.16	0.341	
Herbivory:Orientation	1	62	0.22	0.640	
Month:Herbivory:Orientation	7	62	0.22	0.979	

***Dictyota* spp.**

	num DF	den DF	F	*p*	
(Intercept)	1	62	845.96	<0.001	
Month	7	62	3.12	0.007	**
Herbivory	1	62	19.55	<0.001	***
Orientation	1	62	0.21	0.650	
Month:Herbivory	7	62	2.14	0.052	
Month:Orientation	7	62	4.79	<0.001	***
Herbivory:Orientation	1	62	0.07	0.797	
Month:Herbivory:Orientation	7	62	0.38	0.911	

Articulated calcareous algae

	num DF	den DF	F	*p*	
(Intercept)	1	62	4601.67	<0.000	
Month	7	62	24.01	<0.001	***
Herbivory	1	62	54.02	<0.001	***
Orientation	1	62	27.56	<0.001	***
Month:Herbivory	7	62	8.00	<0.001	***
Month:Orientation	7	62	6.07	<0.001	***
Herbivory:Orientation	1	62	7.88	0.007	**
Month:Herbivory:Orientation	7	62	1.78	0.108	

Sediment

	num DF	den DF	F	*p*	
(Intercept)	1	62	3096.64	0.000	
Month	7	62	13.90	<0.001	***
Herbivory	1	62	0.19	0.662	
Orientation	1	62	333.38	<0.001	***
Month:Herbivory	7	62	2.45	0.028	*
Month:Orientation	7	62	12.76	<0.001	***
Herbivory:Orientation	1	62	0.06	0.808	
Month:Herbivory:Orientation	7	62	2.68	0.017	*

Turf associated with sediment (TAS)

	num DF	den DF	F	*p*	
(Intercept)	1	62	10718.23	<0.001	
Month	7	62	106.78	<0.001	***
Herbivory	1	62	13.11	0.001	***
Orientation	1	62	414.09	<0.001	***
Month:Herbivory	7	62	9.89	<0.001	***
Month:Orientation	7	62	104.99	<0.001	***
Herbivory:Orientation	1	62	11.94	0.001	***
Month:Herbivory:Orientation	7	62	9.93	<0.001	***

Analysis of algal community composition on experimental substrates - PERMANOVA

PERMANOVA across time

	Df	SumsOfSqs	MeanSqs	F.Model	R^2	*p*	
Month	7	15.29	2.18	18.26	0.240	0.010	**
Herbivory	1	1.32	1.31	11.00	0.021	0.010	**
Orientation	1	12.38	12.28	102.63	0.192	0.010	**
Month:Herbivory	7	3.22	0.50	3.85	0.051	0.010	**
Month:Orientation	7	10.31	1.47	12.31	0.161	0.010	**
Herbivory:Orientation	1	0.41	0.41	3.41	0.006	0.020	*
Month:Herbivory:Orientation	7	1.79	0.26	2.14	0.028	0.010	**
Residuals	160	19.14	0.12		0.300		
Total	191	63.74			1.000		
