# Supplementary material for: Interactive effects of herbivory and substrate orientation on algal community dynamics on a coral reef
Source: Mar Biol. 2018 Sep 14;165(10):156. doi: 10.1007/s00227-018-3411-2 (PMC6153878; doi:10.1007/s00227-018-3411-2)
Supplement: Supplementary file 2 — Supplementary material 2 (PDF 672 kb) [file 227_2018_3411_MOESM2_ESM.pdf]

Title: Interactive effects of herbivory and substrate orientation on algal community dynamics on a coral reef

Authors: A. Duran, L. Collado-Vides, L. Palma, and D. E. Burkepile

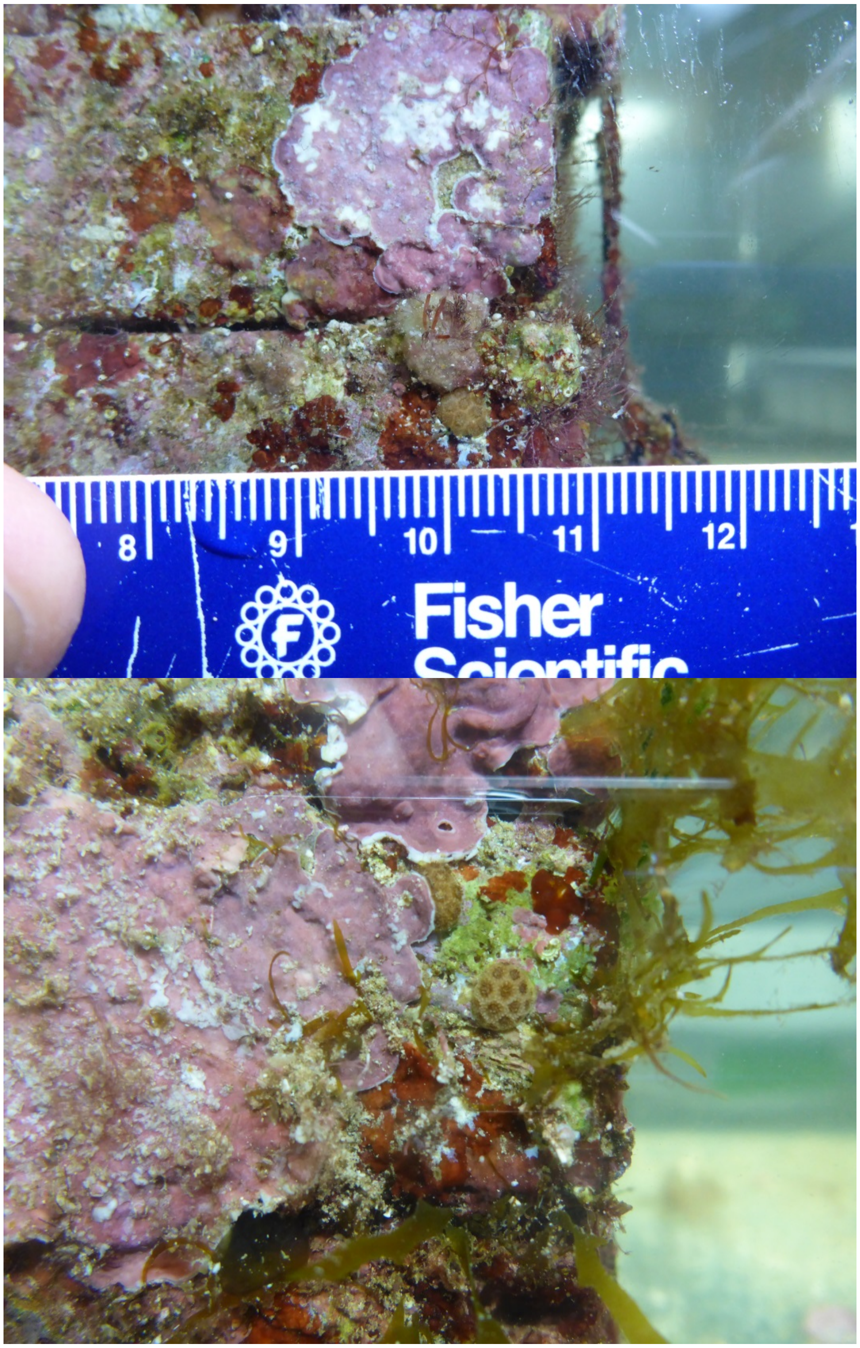

Figure 1. Photographs of the two juvenile *Porites* spp. coral recruits found on vertical substrates. We found no recruits on horizontal substrates.
